# Parenting Styles, Prosocial, and Aggressive Behavior: The Role of Emotions in Offender and Non-offender Adolescents

**DOI:** 10.3389/fpsyg.2017.01246

**Published:** 2017-08-09

**Authors:** Anna Llorca, María Cristina Richaud, Elisabeth Malonda

**Affiliations:** ^1^Department of Personality, Evaluation and Psychological Treatment, University of Valencia Valencia, Spain; ^2^National Council of Scientific and Technological Research Buenos Aires, Argentina; ^3^Basics Psychology, University of Valencia Valencia, Spain

**Keywords:** parenting styles, aggression, prosocial behavior, emotional instability, empathy, non-offender adolescents, offender adolescents

## Abstract

The aim is to analyse the parenting styles effects (acceptance, negative control and negligence) on prosociality and aggressive behavior in adolescents through the mediator variables empathy and emotional instability, and also, if this model fits to the same extent when we study adolescents institutionalized due to problems with the law and adolescents from the general population, and at the same time, if the values of the different analyzed variables are similar in both groups of adolescents. We carried out a cross-sectional study. 220 participants from schools in the metropolitan area of Valencia took part in the study. Also, 220 young offenders took part recruited from four Youth Detention Centres of Valencia, in which they were carrying out court sentences. The age of the subjects range from 15-18 years. The results indicate that the emotional variables act as mediators in general, in the non-offender adolescents, but it has been observed, in the offender adolescents, a direct effect of support on aggressive behavior in a negative way and on prosociality in a positive way; and of negligence on aggressive behavior and of permissiveness on prosociality in a negative way.

## Introduction

Due to its relevance, there has been an increase in interest to research prosocial development in childhood and adolescence in recent years, in particular, as a moderator factor of aggressive behavior and as a disposition that encourages social adaptation.

A large number of studies have demonstrated the importance of parenting style in the transmission of values and in the encouragement of prosocial behaviors (Carlo et al., 2010; Richaud de Minzi et al., 2011). Indeed, parental support predicts a strong sense of self-worth and security, greater psychological well-being, and other positive outcomes (Steinberg, 2001; Coplan et al., 2002). Parental control helps to shape responsible conformity and self-control in children. The rules and guidelines parents set and enforce teach children about group and societal standards of behavior (Baumrind, 1966). Maccoby and Martin (1983), distinguish different types “styles of parenting” based on the balance between high and low levels of parental responsiveness (i.e., support) and demands (i.e., control). One of them is authoritative parents, which display high levels of both responsiveness and demands. This parents are warm, nurturing, and sensitive to their child's needs and consistently consider the child's age and maturity when forming behavioral expectations (Rothrauff et al., 2009). Children who exhibit higher levels of prosocial behavior generally have authoritative parents (high support, high demans) rather than authoritarian parents (low support, high demands) or neglectful ones (low support, low demands; Baumrind, 1991; see Maccoby and Martin, 1983). Parents might coach and guide their children's prosocial behaviors by providing direct verbal messages (e.g., beliefs, attitudes) about desirable behaviors (Carlo, 2006).

Conversely, numerous studies have manifested that negative praxis from the parents, like excessive control and extreme permissiveness, perceived by the child as negligence and ignorance on the part of the parents when seeing to their needs, have a negative effect in the emotional development of the children, prompting in part, behavioral problems and aggressive behavior (e.g., Eisenberg et al., 2000; Samper et al., 2008; Gámez-Guadix et al., 2010; Mestre et al., 2010; Richaud, 2010; Calvete et al., 2014; Llorca-Mestre et al., 2017b). In reference to this, Doyle et al. (2004) state that the quality of the parent-child relationship became an important predictor in the adjustment of the child in mid and late childhood. Children who have good relationships with their parents are less inclined to experience indirect or manifest aggression, upset others or get involved with deviant peers (Mestre et al., 2007; Calvete et al., 2014). This children are more involved in their school work, have a higher self-esteem and less internalized problems.

Chao and Willms (2002) found that positive praxis from the parents (sensitive, rational, strong parenting) have positive effects in the results of the children; reducing the levels of behavioral problems and increasing prosocial behavior (Padilla-Walker et al., 2012; Abar et al., 2014; Mestre, 2014; Grusec and Hastings, 2015; Pastorelli et al., 2016).

Despite the evidence of the relations between parenting styles and prosocial behavior, these results are quite scarce, especially among adolescents (Carlo et al., 2007). This could be do to the fact that the parents' style is a combination of attitudes toward the child which together create an emotional climate (Darling and Steinberg, 1993; Grusec, 2011); these attitudes don't express specific behaviors in particular situations but a frame in which to be developed, furthermore, their influence with regards to the development of behavior like prosociality or aggressive behavior is non-defining. Therefore, we think that parental styles are probably related to prosociality but mediated by emotional aspects like empathy and emotional instability.

Empathy in adolescents is greatly influenced by early experiences of interpersonal relationships. During childhood caregivers influence emotional development to the extent that they provide stimuli for emotions at appropriate times, reinforce and stimulate emotional expression and respond to subtle variations on the children's expressions. There is a substantial body of literature that is consistent with the conclusion that both the general tone of parenting and specific parenting practices are related to the development of empathy and sympathy (Eisenberg et al., 2010). Previous studies have found that empathy in adolescents is explained by empathic feelings that the adolescents perceive in their parents (Richaud de Minzi, 2013). Parents can stimulate compassionate empathic activity by shaping empathic worry and using ways to discipline with an affective orientation to help children understand the harming effects of causing distress to others (Mestre et al., 2007). Empathy involves not only the affective experience of the real or inferred emotional state of another, but also a small measure of recognition and comprehension of another's emotional state (Decety et al., 2008).

On the other hand, Eisenberg et al. (2010) conclude that empathy and/or sympathy seem to play a role in the degree to which individuals engage in other-oriented prosocial behavior and antisocial behavior. There is evidence that empathic individuals are less aggressive due to their emotional sensitivity and their ability to understand the potential negative consequences to self and others that can result from aggressive behavior (see among others Miller and Eisenberg, 1988; Maibom, 2012). At the same time, other studies have shown a direct relationship between empathy and prosocial behavior (Carlo et al., 2011; Richaud de Minzi et al., 2011; Geng et al., 2012; Panfile and Laible, 2012; Sahdra et al., 2015).

It has also been observed that an inadequate emotional development often leads to irritable and impulsive children with little control, prone to externalizing behavioral problems in childhood or later in adolescence or adulthood, who can exhibit dysfunctional behavior and even break the law (Bandura, 1999; Eisenberg et al., 2000; Caprara et al., 2010; Justicia and Cantón, 2011; Mestre et al., 2012; Simone et al., 2012; McMahon et al., 2013; Llorca-Mestre et al., 2017a).

Indeed, Jolliffe and Farrington (2004) found that cognitive empathy had a stronger negative relation with delinquency than did affective empathy, regardless of the type of offense or the age group studied. However, when comparing the relation of empathy with breaking the law in adults vs. adolescents, they found a more consistent negative relation of delinquency with affective empathy (but not cognitive empathy) for adolescents compared to adults (Eisenberg et al., 2010).

Given this negative correlation between empathy and delinquency and, at the same time, the emotional instability with transgressor behaviors, we were interested in studying the model of the relation between parenting, emotional development and prosocial and aggressive behavior in adolescents who have problems with the law.

In line with this, Hoeve et al. (2009) found, in accordance with the finding of Loeber and Stouthamer-Loeber (1986), that parental rejection and poor supervision were among the best predictors of delinquency. In particular, a neglectful parenting style may be linked to delinquency (Maccoby and Martin, 1983; Steinberg et al., 1994). Poor parental monitoring was also relatively strongly linked to delinquency. The three indicators of parental monitoring, that is, parental knowledge of the child's whereabouts, the active tracking and tracing of the child's whereabouts by parents, and child disclosure, had links to delinquency that were relatively similar in magnitude.

According to these theoretical and empirical antecedents, the aim of the present study is to analyse the parenting styles effects (acceptance, negative control and negligence) on prosociality and aggressive behavior in adolescents through the mediator variables empathy and emotional instability, and also, if this model fits to the same extent when we study adolescents institutionalized due to problems with the law and adolescents from the general population, and at the same time, if the values of the analyzed variables are similar or different in both groups of adolescents.

## Materials and methods

### Participants

(1) 220 participants randomly selected from 10 public and private schools in the metropolitan area of Valencia (Spain) took part in the study. As for the sex of the participants there were 145 boys (65.9%) and 75 girls (34.1%). The age of the subjects range from 15 to 18 years, giving a mean age of 16.40 with a standard deviation of 1.25. As for the social class of the studied adolescents we observe that for the most part they come from families of social class III or middle class (35.9%) and social class IV or low-middle class (37.7%). We find to a lesser degree families of social class II or upper-middle class (11.8%) and social class V or lower class (8.2%) (Hollingshead, 1975).

(2) 220 young offenders took part recruited from four Youth Detention Centres of Valencia (Spain), in which they were carrying out court sentences. Among the crimes this youngsters were carrying out different court sentences for, violence against their parents, damage against property, public health crimes and bodily harm stand out. With regards to the sex of the participants in the offender sample we find a total of 148 boys (67.3%) and 72 girls (32.7%). In the institutionalized boys and girls we find a mean age of 16.22 and a standard deviation of 1.49. If we consider the crime committed that has originated the stay in the Centre for Minors, it is verified that the more dominant one is child to parent violence (60.7%) followed by aggravated robbery (33.7%) and in a lesser degree other crimes are attempt against authority (2.6%), breach of parole (2%) and bodily harm (1%).

With regards to social class of the adolescents who are carrying out court sentences, most of the families are of social class IV or lower middle class (51.4%), followed by social class III or middle class (23.2%) and to a lesser degree we find families that belong to a social class II upper middle class (3.2%) and a social class V or lower class (6.8%).

In general, Spain is an Occidental society, developed and industrialized (Andreu, 2014). Spain is characterized as a society that values family, in fact, un 85.4% of the general population values it as very important after health with a 88.4% (CIS, 2014). Moreover, family is seen as being more important than country, religion, or politics (Sánchez and Bote, 2009). In the Spanish population, researchers have observed mothers' and fathers' tendency toward similar socialization styles for boys and girls from 11 to 17 years (Garaigordobil and Aliri, 2011). However, according to data from the INJUVE (2012), girls from 15 to 24 years old feel more protection and parental control, and less parental permissiveness than boys feel.

### Procedure

We carried out a cross-sectional study. The participants have filled in self-assessment questionnaires. In the schools the instruments were applied collectively in the classroom for about 50 min. The study was presented to the teachers of the schools, the authorisation of the Valencian Government was obtained and written informed consent was obtained from the parents of the participants under that age of 16. The participation of the adolescents was voluntary and anonymous, taking into consideration all the ethical principles pertaining to studies carried out on human beings included in the Helsinki Declaration, under current regulations.

In the youth detention centers the application of the questionnaires was carried out in small groups made out of two or three and when necessary they were carried out individually with the help of trained professionals. The research project was presented to the management of the youth detention centers in Valencia that took part in the study. The cooperation of the centers and the evaluation carried out had the authorisation of the Valencian Government and written informed consent was obtained from the parents of the participants under that age of 16. The participation of the adolescents was voluntary and anonymous, taking into consideration all ethical principles pertaining to research with human beings included in the Helsinki Declaration, under the current regulations. The research project had a favorable response from the university ethics committee because it is required for the concession of these studies (GVPROMETEO/2015/003 and PSI2016-78242-R).

### Instruments

#### Physical and verbal aggression scale (PVA, Caprara and Pastorelli, 1993; del Barrio et al., 2001)

It evaluates behaviors that harm others physically or verbally. It is made up of 20 items with three response choices (often, sometimes or never). Sample item: “I speak badly of my peers.” The Cronbach's Alpha was 0.90.

#### Emotional instability scale (IE, Caprara and Pastorelli, 1993; del Barrio et al., 2001)

It describes the behavior that indicates lack of self-control in social situations as a result of the limited ability to curb impulsiveness and emotionality. It is made up of 15 items with three response choices (often, sometimes or never). Sample item: “I interrupt others when they talk”. Cronbach's Alpha was 0.85.

#### Prosocial behavior CP, Caprara and Pastorelli 1993; del Barrio et al., 2001)

*Prosocial Behavior* (CP, Caprara and Pastorelli 1993; del Barrio et al., 2001) evaluates helping behavior, trust and sympathy. It is made up of 15 items with three response choices (often, sometimes or never), depending on how often the participant gets involved in a particular conduct. Sample item: “I try to help others.” Cronbach's Alpha was 0.81.

#### Child reports of parental behavior inventory (Schaefer, 1965; CRPBI, Samper et al., 2006)

This questionnaire uses 38 items to evaluate the parenting styles, distinctly for their father and mother, which establish parent-child relationships from the point of view of the adolescent. There are 3 possible answers: 1 (never), 2 (sometimes), and 3 (always). The instrument is made out of four factors. Support and communication refers to the perception of emotional support and affection perceived by the adolescents, together with the respect for previously established rules. Sample item; “He or she likes to talk about the news with me.” Cronbach Alpha was 0.94. Psychological control, dealing with intrusive control and a negative evaluation of the children. Sample item: “He or she wants to control everything I do.” The Cronbach Alpha was 0.98. Permissiveness is directed to the tendency of the parents to allow the child to do whatever they want without rules or limits. Sample item “He or she lets me go out whenever I want.” The Cronbach Alpha was 0.75. Finally, negligence refers to lack of control and indifference from the parents toward the needs of the adolescents. Sample item: “He or She forgets to give me what I need.” The Cronbach Alpha was 0.79.

### Analyses plan

First of all, Pearson's correlations were carried out among the variables under study with the aim to observe the degree of relation and the relational tendency among them, as well as to establish possible collinear problems among them. Second, MANOVA was carried out to examine whether there were differences in levels of the different variables under study. Finally, the fit of the theoretical method designed through Structural Equation Models (SEM) has been tested in AMOS 17.0 (SPSS Inc, 2007). The following robust statistics have been used to determine the goodness of fit: the chi-squared compared with the degrees of freedom (χ^2^/gl), the robust comparative fit index (CFI robust comparative fit index) the goodness fit index (GFI), the adjusted goodness fit index (AGFI) and the Root mean residual (RMR) (Bollen, 1989).

## Results

### Descriptive statistics

Table 1 shows the correlations among the studied variables in non-offender and offender adolescents. As can be observed, in non-offender adolescents the support of the mother and the father relate negatively to aggressive behavior and emotional instability and positively to prosocial behavior and empathic concern. As for negative control from both parents relates positively to aggressive behavior and emotional instability. Negligence from the parents correlates in a positive way to aggressive behavior in both parents and in a negative way with empathic concern only in the case of the mother.

**Table 1:** Correlations between different variables in non-offender and offender adolescents.

**General Sample**												
**Support Father 1**	**Support father**	**Support mother**	**Negative control father**	**Negative control mother**	**Negligence father**	**Negligence mother**	**Permissiveness father**	**Permissiveness mother**	**Aggressive behavior**	**Prosocial behavior**	**EI**	**Empathic concern**
Support Mother	0.63^**^	1	0.12	0.26^**^	−0.02	0.00	−0.03	0.09	−0.16^*^	0.12	−0.08	−0.01
Negative Control F	−0.26^**^	−0.16^*^	1	0.46^**^	0.28^**^	0.15^*^	−0.09	0.01	0.08	0.05	0.08	0.15^*^
Negative Control M	−0.10	−0.13	0.62^**^	1	0.07	0.25^**^	−0.02	0.04	−0.02	0.00	−0.03	0.09
Negligence Father	−0.26^**^	−0.16^*^	0.40^**^	0.35^**^	1	0.48^**^	0.16^*^	0.15^*^	0.12	−0.06	0.04	−0.04
Negligence Mother	−0.23^**^	−0.33^**^	0.27^**^	0.33^**^	0.58^**^	1	0.10	0.20^**^	0.15^**^	−0.12	0.10	−0.12
Permissiveness Father	0.04	0.09	−0.05	0.09	0.15^*^	0.13	1	0.45^**^	0.06	−0.21^**^	−0.01	−0.17^*^
Permissiveness Mother	0.01	0.07	0.01	0.00	0.17^*^	0.17^*^	0.68^**^	1	0.05	−0.14^*^	0.02	−0.11
Aggressive Behavior	−0.29^**^	−0.23^**^	0.12	0.17^*^	0.14^*^	0.13	0.10	0.06	1	−0.31^**^	0.69^**^	−0.15^*^
Prosocial Behavior	0.16^*^	0.08	−0.13	−0.05	−0.07	−0.09	0.12	0.03	−0.25^**^	1	−0.08	0.36^**^
Emotional Instability	−0.23^**^	−0.16^*^	0.19^**^	0.14^*^	0.10	0.04	0.12	0.16^*^	0.63^**^	−0.07	1	−0.08
Empathic Concern	0.19^**^	0.20^**^	−0.04	−0.12	−0.09	−0.19^**^	−0.05	−0.08	−0.29^**^	0.45^**^	−0.18^**^	1

*Intercorrelations for non-ofender adolescents are presented above the diagonal, and intercorrelations for ofender adolescents are presented below the diagonal*.

* *p < 0.05*;

** *p < 0.01*.

In reference to permissiveness only the permissiveness of the mother relates in a positive way with emotional instability. As for aggressive behavior, it relates negatively to prosocial behavior and empathic concern and positively to emotional instability. With regards to prosocial behavior the relation that stands out is to empathic concern, while emotional instability relates negatively to empathic concern.

In the case of offender adolescents, only the support of the father relates positively to prosocial behavior and only the support of the mother relates negatively to aggressive behavior. As for negative control, it shows no correlation to any of the other variables. As for negligence, only the mother's negligence relates positively to aggressive behavior while permissiveness of both parents relates negatively to prosocial behavior and only the father's permissiveness relates positively to emotional instability.

#### Comparison of the dimensions parental support, negative control, negligence and permissiveness and the variables empathic concern, emotional instability, aggressive behavior, and prosocial behavior between offender and non-offender adolescents.

In order to examine whether there were differences in levels of the different dimensions of parental dimensions (support, negative control, negligence, and permissiveness), a MANOVA was carried out (see Table 2). Also, one-way ANOVAs were carried out to study the differences between means of empathic concern, emotional instability, aggressive behavior and prosocial behavior between offender and non-offender adolescents.

**Table 2:** MANOVAs, Means, standard deviations, MANOVA, and student's *t*-tests for non-offender and offender adolescents.

		**T1**			
		***M***	***DT***	***F***	**$\eta_{p}^{2}$**
Support Father	Non-offenders	2.15	0.42	8.10^**^	0.02
	Offenders	2.02	0.47		
Support Mother	Non-offenders	2.01	0.44	1.84	0.00
	Offenders	1.96	0.46		
Negative	Non-offenders	1.73	0.39		
Control F	Offenders	2.00	0.47	42.10^***^	0.09
Negative	Non-offenders	1.67	0.38	19.49^***^	0.04
Control M	Offenders	1.85	0.44		
Negligence	Non-offenders	1.52	0.47	7.14^**^	0.02
Father	Offenders	1.66	0.61		
Negligence	Non-offenders	1.55	0.43	1.21	0.00
Mother	Offenders	1.60	0.54		
Permissiveness	Non-offenders	1.51	0.50	23.36^***^	0.05
Mother	Offenders	1.77	0.62		
Permissiveness	Non-offenders	1.55	0.50	4.05*	0.01
Mother	Offenders	1.66	0.54		
				***t***	
Aggressive Behavior	Non-offenders	1.32	0.30	9.59^***^	
	Offenders	1.64	0.39		
Prosocial	Non-offenders	2.51	0.32	3.69^***^	
Behavior	Offenders	2.37	0.39		
Emotional	Non-offenders	1.67	0.35	7.80^***^	
Instability	Offenders	1.93	0.36		
Empathic	Non-offenders	3.49	0.62	3.24^**^	
Concern	Offenders	3.31	0.60		

*F, Statistics based on one-way MANOVAs; $\eta_{p}^{2}$, Partial Eta squared, effect size measure (0.01 = small effect; 0.06 = medium effect; 0.13 = large effect; Cohen, 1988)*.

** *p < 0.01*;

*** *p < 0.001*.

*t, Student's t-test*.

MANOVA results show differences in parenting dimensions between non-ofender and offender adolescent [Hotelling's trace criterion *F*_(8, 431)_ = 9.766, *p* ≤ 0.000, η^2^ = 0.15]. On the other hand, results of the univariate analysis indicates that there are statistically significant differences in father support, father negative control, mother negative control, father negligence, father and mother permissiveness. The *t*-tests results show that there are statistically significant differences in aggressive behavior, emotional instability, empathic concern and prosocial behavior (see Table 2).

### Structural equation model. comparison of the model between the two groups of adolescents

A multi-group analysis was used to study if the models didn't have invariance through the offender and non-offender adolescent. For each model, a series of nested models were analyzed and compared by examining the change in model χ^2^ and comparative fit index (CFI) values.

**Figure 1:**
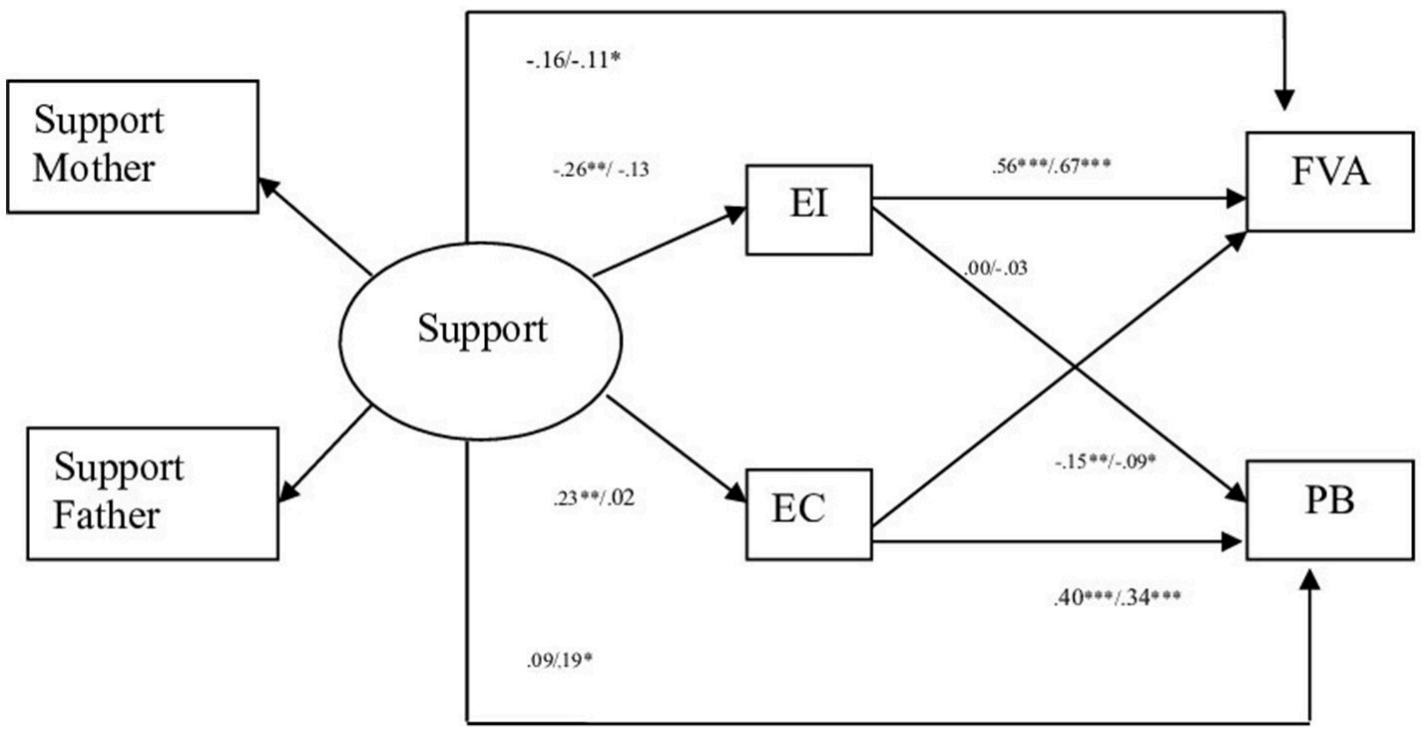
Path standardized coefficient values of offenders and non-offenders pertinent to parental style support. Standardized Values. EI, Emotional Instability; EC, Empathic Concern; PVA, Physical and Verbal Aggressive Behavior; PB, Prosocial behavior. ^***^p < 0.001, ^**^p < 0.01, ^*^p < 0.05. Non offenders left value, offenders right value.

In the first model (dimension parental Support), the comparison of the models resulted in non-significant statistical differences in the χ^2^ for Model 1 (Unconstrained) vs. Model 2 (Measurement weights) and Model 3 (Structural weights) vs. Model 4 (Structural covariances). However, the models resulted in statistically significant χ^2^ differences for Model 2 vs. Model 3, Model 4 vs. Model 5 (Structural residuals) and Model 5 vs. Model 6 (Measurement residuals). The χ^2^ difference tests could be influenced by the sample sizes and its underlying assumption that the model fits the sample data perfectly has long been recognized as problematic (Jöreskog and Sörbom, 1996; Milfont and Fischer, 2010; Kline, 2015). Several fit indexes have thus been developed to overcome limitations of the χ^2^ difference. For example, Cheung and Rensvold (2002) suggest that a difference of CFI of less than or equal to 0.01 is an indicator that the constrained parameters are invariant. However, Milfont and Fischer (2010) suggest that configural invariance, metric invariance and scalar invariance are necessary to compare scores across groups and all additional tests, as error variance invariance is optional. Consequently, these results provide useful information about the stability of the model though offender and non-offender adolescents. The results indicated that the theoretical model 1 (dimension parental Support) fit equally well for offender and non-offender adolescents (see Table 3). The standardized coefficients show that non-offenders parental support relates positively to empathic concern and negatively to emotional instability. At the same time, empathic concern relates positively to prosocial behavior and negatively to aggressive behavior, and emotional instability has a direct relation to aggressive behavior (see Figure 1). Respecting the offenders, the standardized coefficients show that support doesn't relate to empathic concern nor emotional instability, but shows a direct and negative relation to aggressive behavior and a direct and positive relation to prosociality. At the same time, empathic concern relates positively to prosocial behavior and negatively to aggressive behavior and emotional instability while emotional instability has a direct relation to aggressive behavior. Finally, a direct relation exists between parental support and prosocial behavior (see Figure 1).

**Table 3:** Comparative indexes for first model (dimension parental support).

	**χ^2^**	**df**	***p***	**χ^2^/ df**	**GFI**	**AGFI**	**CFI**	**RMR**	***Δχ*^2^**	***Δχ*^2^/df**	**ΔCFI**
Model 1	42.148	10	0.00	4.215	0.97	0.88	0.94	0.008			
Model 2	48.292	15	0.00	3.219	0.96	0.91	0.93	0.011	6.14	5	
Model 3	60.503	19	0.00	3.184	0.96	0.91	0.92	0.012	12.21	4	0.01
Model 4	61.832	20	0.00	3.092	0.96	0.90	0.92	0.014	1.329	1	0.01
Model 5	95.122	26	0.00	3.659	0.93	0.89	0.87	0.017	33.29	6	0.00
Model 6	593.422	30	0.00	19.781	0.70	0.58	0.00	0.042	498.3	4	0.05

*Model 1 (Unconstrained) vs. Model 2 (Measurement weights) and Model 3 (Structural weights) vs. Model 4 (Structural covariances)*.

**Figure 2:**
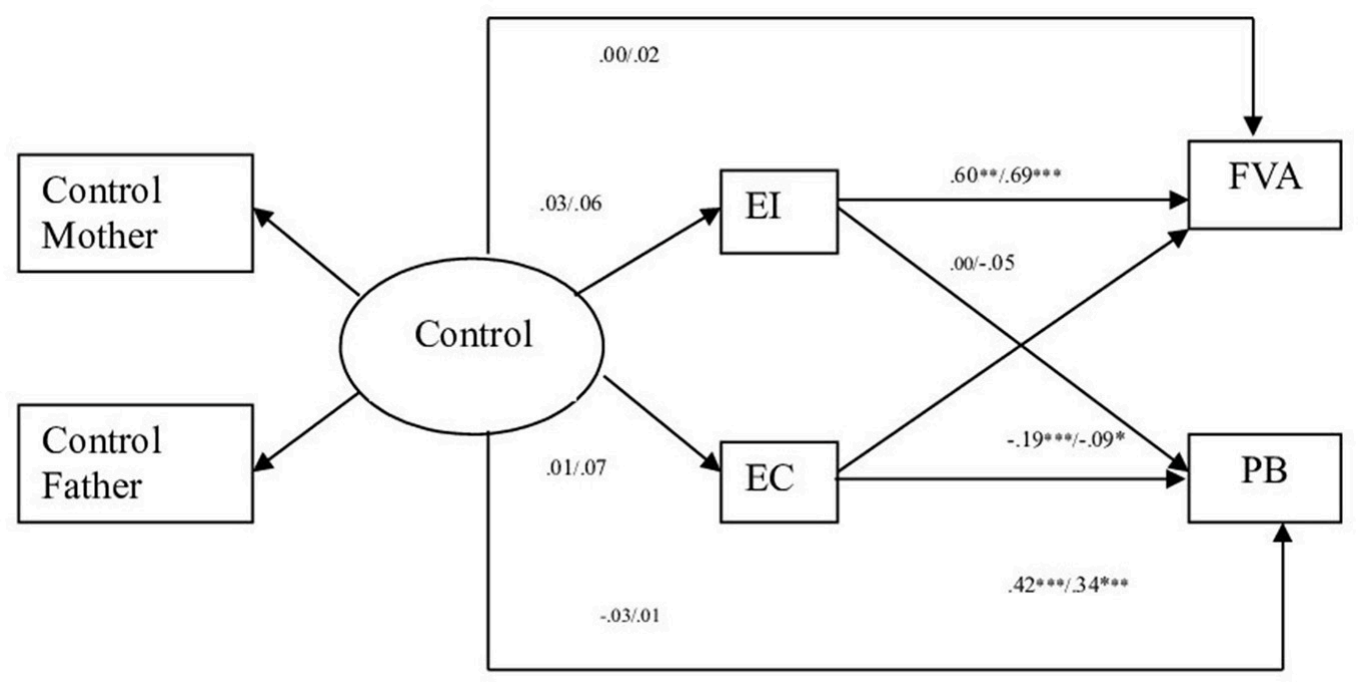
Path standardized coefficient values of offenders and non-offenders pertinent to parental style negative control. Standardized Values. EI, Emotional Instability; EC, Empathic Concern; FVA, Physical and Verbal Aggressive Behavior; PB, Prosocial behavior. ^***^p < 0.001, ^**^p < 0.01, ^*^p < 0.05. Non offenders left value, offenders right value.

In the second model (dimension parental Negative Control), the comparison of the models resulted in non-significant statistical differences in the χ^2^ for Model 2 (Measurement weights) and Model 3 (Structural weights) vs. Model 4 (Structural covariances). The differences between the CFI values were the same or less than 0.01. The results indicated then that the theoretical model II (dimension parental Negative Parental) fit equally well for offender and non-offender adolescents (see Table 4). The standardized coefficients in non-offender-adolescents only show significant relations in empathic concern, which relates positively to prosocial behavior and negatively to aggressive behavior, and emotional instability, which relates directly to aggressive behavior (see Figure 2). Concerning offender adolescents, the standardized coefficients only show significant relations in empathic concern, which relates positively to prosocial behavior and negatively to aggressive behavior; and in emotional instability that has a direct relation to aggressive behavior (see Figure 2).

**Table 4:** Comparative indexes for second model (dimension parental negative control).

	**χ^2^**	**df**	***p***	**χ^2^/df**	**GFI**	**AGFI**	**CFI**	**RMR**	***Δχ*^2^**	***Δχ*^2^/df**	**ΔCFI**
Model 1	4.804	10	0.00	4.880	0.96	0.85	0.93	0.013			
Model 2	53.845	15	0.00	3.590	0.96	0.89	0.93	0.014	49.04	5	
Model 3	64.894	19	0.00	3.415	0.95	0.90	0.92	0.015	11.04	4	0.00
Model 4	64.899	20	0.00	3.245	0.95	0.90	0.92	0.015	0.005	1	0.01
Model 5	109.583	26	0.00	4.215	0.92	0.87	0.85	0.019	44.684	6	0.00
Model 6	571.722	30	0.00	19.057	0.72	0.62	0.00	0.038	462.14	4	0.07

*Model 1 (Unconstrained) vs. Model 2 (Measurement weights) and Model 3 (Structural weights) vs. Model 4 (Structural covariances)*.

**Figure 3:**
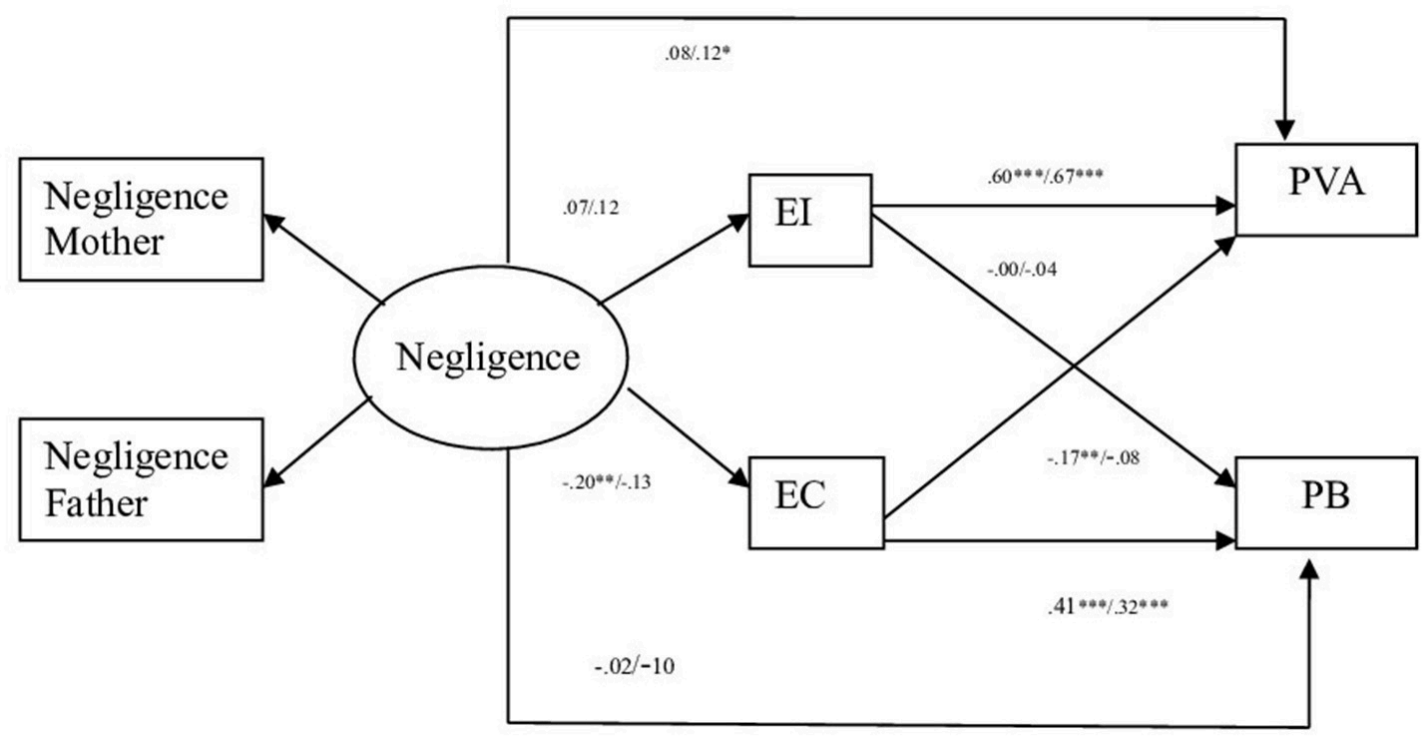
Path standardized coefficient values of offenders and non-offenders pertinent to parental style negligence. Standardized Values. EI, Emotional Instability; EC, Empathic Concern; PVA, Physical and Verbal Aggressive Behavior; PB, Prosocial behavior. ^***^p < 0.001, ^**^p < 0.01, ^*^p < 0.05. Non offenders left value, offenders right value.

In the third model (dimension parental Negligence), the comparison of the models resulted in non-significant statistical differences in Model 1 (Unconstrained) vs. Model 2 (Measurement weights) and Model 3 (Structural weights) vs. Model 4 (Structural covariances). The differences between the CFI values were the same or less than 0.01. The results indicated then that the theoretical model II (dimension parental Negligence) fit equally well for offender and non-offender adolescents (see Table 5). The standardized coefficients show that non-offenders parental negligence relates negatively to empathic concern. At the same time, empathic concern relates positively to prosocial behavior and negatively to aggressive behavior and emotional instability relates directly to aggressive behavior (see Figure 3). The standardized coefficients corresponding to offender adolescents, show that parental negligence does not relate to empathic concern or emotional instability, but it shows a direct relation to aggressive behavior. At the same time, empathic concern relates in a positive way only to prosocial behavior, and emotional instability shows a direct relation to aggressive behavior (see Figure 3).

**Table 5:** Comparative indexes for third model (dimension parental neglience).

	**χ^2^**	**Df**	***p***	**χ^2^/df**	**GFI**	**AGFI**	**CFI**	**RMR**	**Δχ^2^**	**Δχ^2^/df**	**ΔCFI**
Model 1	41.526	10	0.00	4.15	0.97	0.88	0.94	0.010			
Model 2	43.229	15	0.00	2.88	0.97	0.91	0.94	0.010	1.70	5	
Model 3	53.704	19	0.00	2.83	0.96	0.92	0.93	0.012	10.47	4	0.00
Model 4	56.194	20	0.00	2.81	0.96	0.92	0.93	0.014	2.49	1	0.01
Model 5	102.648	26	0.00	3.95	0.93	0.88	0.85	0.025	46.45	6	0.00
Model 6	552.840	30	0.00	18.42	0.73	0.61	0.00	0.045	450.19	4	0.08

*Model 1 (Unconstrained) vs. Model 2 (Measurement weights) and Model 3 (Structural weights) vs. Model 4 (Structural covariances)*.

**Figure 4:**
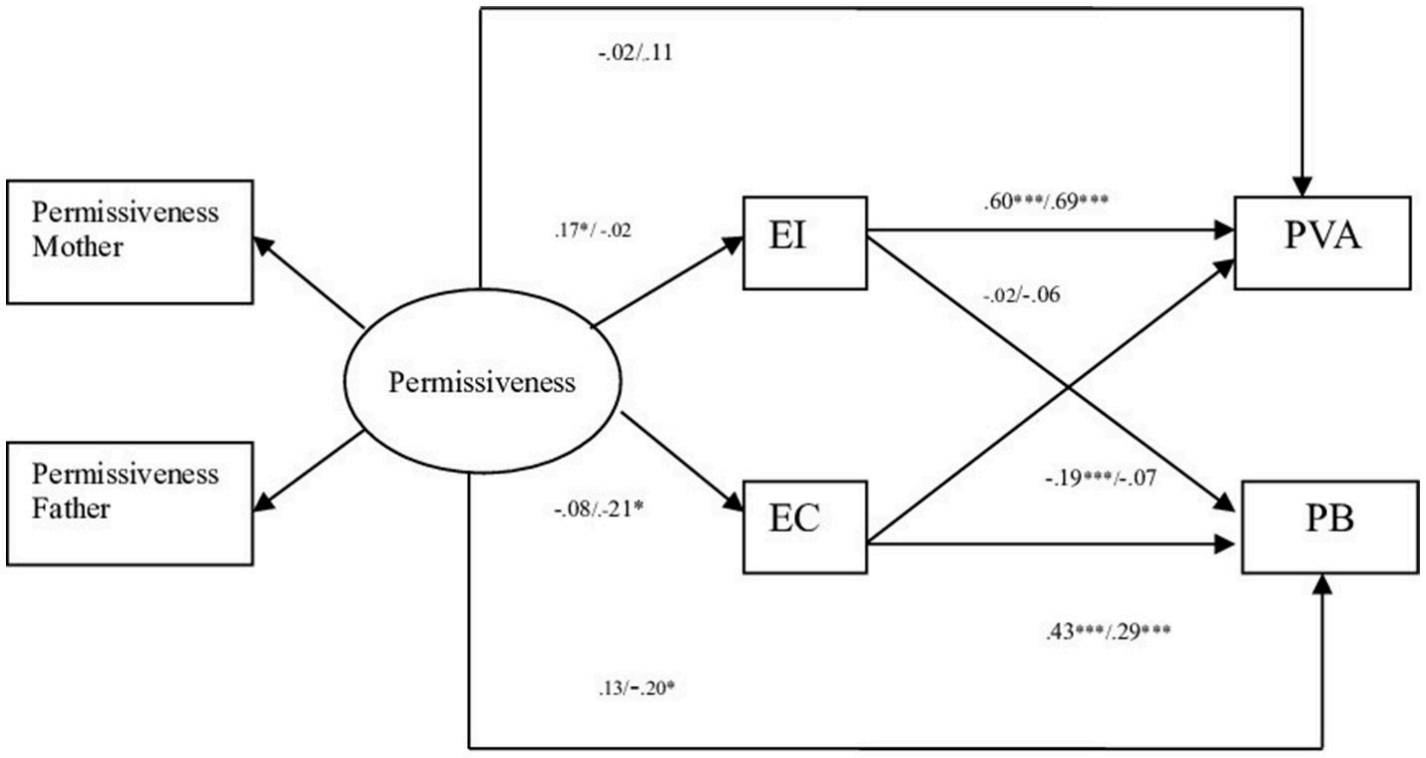
Path standardized coefficient values of offenders and non-offenders pertinent to parental style permissiveness. Standardized Values. EI, Emotional Instability; EC, Empathic Concern; PVA, Physical and Verbal Aggressive Behavior; PB, Prosocial behavior. ^***^p < 0.001, ^*^p < 0.05. Non offenders left value, offenders right value.

In the fourth model (dimension parental Permissiveness), the comparison of the models resulted in non-significant statistical differences in the χ^2^ for Model 3 (Structural weights) vs. Model 4 (Structural covariances). The differences between the CFI values were the same or less than 0.01. The results indicated then that the theoretical model II (dimension parental Permissiveness) fit equally well for offender and non-offender adolescents (see Table 6). In the case of non-offender adolescents, the standardized coefficients show that permissiveness relates positively to emotional instability. At the same time, empathic concern relates positively to prosocial behavior and negatively to aggressive behavior, and emotional instability relates directly to aggressive behavior (see Figure 4). With regards to offender adolescents, the standardized coefficients show that permissiveness relates negatively to empathic concern and in a negative direct way to prosociality. At the same time, empathic concern relates positively to prosocial behavior and emotional instability relates directly to aggressive behavior (see Figure 4).

**Table 6:** Comparative indexes for fourth model (dimension parental permissiveness).

	**χ^2^**	**Df**	***p***	**χ^2^/df**	**GFI**	**AGFI**	**CFI**	**RMR**	**Δχ^2^**	**Δχ^2^/df**	**ΔCFI**
Model 1	42.776	10	0.00	4.28	0.97	0.87	0.94	0.009			
Model 2	60.191	15	0.00	4.01	0.96	0.88	0.93	0.014	17.42	5	
Model 3	71.426	19	0.00	3.76	0.95	0.89	0.92	0.015	11.24	4	0.01
Model 4	71.784	20	0.00	3.59	0.95	0.89	0.91	0.016	0.36	1	0.01
Model 5	127.926	26	0.00	4.92	0.91	0.86	0.82	0.024	56.14	6	0.00
Model 6	603.769	30	0.00	20.13	0.72	0.61	0.00	0.05	475.84	4	0.09

*Model 1 (Unconstrained) vs. Model 2 (Measurement weights) and Model 3 (Structural weights) vs. Model 4 (Structural covariances)*.

## Discussion

We have hypothesized that parental styles would have an effect on prosociality and aggressive behavior mediated by empathic concern and emotional instability and that this model remains invariant through non-offender and offender adolescents.

The results partially support this hypothesis since the emotional variables act as mediators in general, in the non-offender adolescents (Carlo et al., 2010; Llorca-Mestre et al., 2017a), but it has been observed, in the offender adolescents, a direct and negative effect of parental support on aggressive behavior and a direct positive effect on prosociality. At the same time, the results indicated a direct and negative effect of parental negligence on offender aggressive behavior and a direct and negative effect of parental permissiveness on prosociality and of negligence on aggressive behavior (Chao and Willms, 2002; Mestre, 2014; Grusec and Hastings, 2015). It would seem that in the young offenders the parental styles would not act on the emotional development that would serve as mediator for aggressive behavior or prosocial behavior, but would lead directly to the action. The opposite occurs in non-offender adolescents where it would seem that the way parents bond with their children would be related to a more functional (empathic concern) or more dysfunctional (emotional instability) emotional development, which would ultimately determine the specific behavior of aggressiveness or prosociality.

In the case of adolescent perception of parental support, only in non-offenders is significantly related to both emotional instability (in a negative way) and empathic concern (in a positive way). That is to say that the perception of support in this case serves on the one hand as a protective factor against emotional instability and on the other as a strengthening factor of empathic concern or concern others (Samper-García et al., 2015). This is not the case with adolescent offenders where parental support, especially from the father, is lower than that of non-offenders, accompanied by greater negligence and permissiveness, which is likely to diminish emotional development, causing the lack of support to directly affect a greater externalization of behaviors such as aggression (Gámez-Guadix et al., 2010; Mestre et al., 2010; Calvete et al., 2014).

In the case of parental negative control, i.e., extreme through punishment, isolation or anxiety production, it would appear to inhibit the emotional development in the two groups of adolescents, not having a relation to either emotional instability or empathic concern (Richaud, 2010).

With regard to parental negligence, i.e., not meeting the needs of children due to lack of interest and affection, presented a direct and significant influence on aggressive behavior in offender adolescents and a significant relationship with emotional instability in non-offender adolescents (Mestre et al., 2010). It would seem then that in the offender adolescents, at least in most of those we have seen, had conflicts with their parents, the perception of lack of affection and interest from them generates aggressive behavior. On the other hand, in non-offenders, negligence is negatively related to empathic concern, as if perceiving that no one cares for them will lead them not to develop interest and concern others.

Finally, adolescent perception of parental permissiveness would lead the non-offenders to greater emotional instability, that is, that the lack of limits, still accompanied by acceptance, would produce in the child mixed feelings and difficulty to regulate themselves (Gámez-Guadix et al., 2010; Mestre et al., 2010; Llorca-Mestre et al., 2017b). In the case of offenders, where we have already said that acceptance is lower, permissiveness is probably perceived as lack of interest, that is, more like negligence, relating to less empathic concern or interest in the other. In all four cases (parental support, negative control and neglect) the expected relationship between emotional instability and aggressive behavior and between empathic concern and prosocial behavior was maintained.

## Conclusions

Even though the model that postulates that the parental style relates to prosociality and aggressive behavior through empathic concern and emotional instability has shown invariance in the offender and non-offender adolescents in the four parental dimensions: support, negative control, negligence and permissiveness, it would seem that in the offenders there has been a lesser emotional development which leads to externalize the behavior in a more direct way.

The perceived parental support would be an important promoter factor for empathic concern and prosociality and a protector against emotional instability and aggressive behavior.

The perceived parental negative control would inhibit emotional development in both offenders and non-offenders.

Parental negligence would be a risk factor in both groups of adolescents, although in the offenders it would encourage aggressive behavior and in the non-offenders the lack of interest in others and therefore prosocial behavior would be reduced.

Parental permissiveness would produce emotional instability in the non-offender adolescents and in the offenders a lack of interest in others.

## Limitations and future directions

A limitation of the present study is its correlational nature that did not allow for the establishment of causal explanations. The study was based on cross-sectional data; therefore, the direction of the effects in the models may not be clear. Being a one-time self-assessment, it could have an inherent method effect contributing to the strength of all of the relationships here studied. Consequently it would be necessary in future research to analyse the relationships analyzed herein, with longitudinal studies.

Finally, the present research was carried out in a specific culture and with adolescents who in their majority (60.7%) showed child to parent violence. Future research will take into account different infractions not contemplated in the present study.

## Ethics statement

All subjects gave written informed consent in accordance with the Declaration of Helsinki. The protocol was approved by the Regional Government of Valencia.

## Author contributions

AL and EM: Materials and Methods, and Results. MCR: Introduction and Discussion.

### Conflict of interest statement

The authors declare that the research was conducted in the absence of any commercial or financial relationships that could be construed as a potential conflict of interest.
